# Adjunctive use of mindfulness-based mobile application in depression: randomized controlled study

**DOI:** 10.1007/s00406-024-01884-y

**Published:** 2024-09-04

**Authors:** Jan Sarlon, Else Schneider, Annette B. Brühl, Sarah Ulrich, Timur Liwinski, Jessica P. Doll, Markus Muehlauser, Undine E. Lang

**Affiliations:** University of Basel, University Psychiatric Clinics (UPK), Basel, Switzerland

**Keywords:** Depression, Mindfulness, Mobile application, Stress level

## Abstract

Mindfulness-based interventions (MBI) are effective in relapse prevention in Major Depressive Disorder (MDD). Internet-based interventions have been demonstrated to be effective in the treatment of MDD. Consequently, the integration of MBI through mobile applications emerges as a promising supplementary intervention for MDD, contributing to the augmentation of mental health services, particularly within ambulatory care contexts. The current randomized controlled study is designed to evaluate the efficacy of adjunctive MBI delivered via a mobile app in mitigating symptom severity and stress levels. This assessment involves a comparison with standard treatment practices in an ambulatory setting among individuals diagnosed with MDD. A total of 83 patients diagnosed with MDD (depressive episode, recurrent depression or depressive phase of bipolar disorder) were randomly allocated to the intervention (41 patients) or control condition (42 patients). The intervention consisted of the daily use of the mindfulness mobile application “Headspace” for thirty days. The control condition was treatment as usual (TAU) only. The symptom severity has been assessed by the Beck Depression Inventory (BDI-II) as well as the Hamilton Depression Rating Scale (HDRS-17). Blood pressure and resting heart rate have been assessed as secondary outcome. Upon hospital discharge, the mean scores on the Beck Depression Inventory (BDI) and Hamilton Depression Rating Scale (HDRS) signaled partial remission of MDD in both treatment arms. In both groups, a subsequent decrease in both self-reported and expert-rated scores was evident after a 30-day period. However, the decrease in depression severity as measured by HDRS was significantly higher in the MBI group compared to the control group after 30 days. For secondary outcomes, systolic blood pressure was lower in the intervention group compared to control group. The total drop-out rate was 29%. Short term mindfulness intervention via mobile application (30 days) can be beneficial as adjunctive therapy to treatment as usual in patients with MDD.

## Introduction

Internet-based interventions for Major Depressive Disorder (MDD) are acknowledged for their efficacy, ease of access and cost-effectiveness. Hence, these interventions have the potential to narrow the treatment gap between the demand and availability of psychiatric treatments for depression by reaching broad and diverse populations [1–4]. Furthermore, internet-based interventions may be an effective option for people with depression who cannot or prefer not to access supervised treatment [5]. A meta-analysis of Serrano-Ripoll assessing app-based interventions demonstrated a moderate reductions in the symptom severity in depression [6]. The authors point out that more data is needed to determine which intervention features are associated with greater improvements, and to identify those populations who most likely benefit from this type of intervention.

Mindfulness-based interventions (MBI)—such as mindfulness-based stress reduction (MBSR) or mindfulness-based cognitive therapy (MBCT) have received considerable attention in the last decades because of emerging evidence regarding their efficacy in the treatment of depression [7–11]. The effects of MBCT on reduction of depressive symptoms and relapse prophylaxis are comparable to those of other cognitive behavioural therapies [12–15] and showed favourable effects, if used as adjunct therapy to a treatment as usual (TAU) [16–19]. A currently ongoing randomized controlled trial aims to confirm the clinical effectiveness of MBCT in depressed non-responders [20].

The mindfulness-based intervention can be delivered via various apps. Within the realm of commercial applications, "Headspace" has been subjected to repeated randomized controlled trials, confirming its effectiveness in evaluating stress levels, mind wandering, irritability, and both positive and negative affect [21–24], making this mobile app promising for further research. Taylor et al. [25] showed small effects of Headspace versus active control (psychoeducational digital platform) for depression, assessed by Depression Anxiety Stress Scale (DASS-21). Ly et al. [26] demonstrated comparable effects of a self-developed mindfulness-based app and behavioural activation via an app major depression on a clinical population of MDD. Nonetheless, research on MBI through mobile applications for depression is limited, thereby constraining the generalizability of conclusions regarding their utility in clinical practice and necessitating further methodologically robust randomized controlled trials.

The aim of present study is, therefore, to examine the effects of a mindfulness-based mobile application (Headspace®) on the symptom severity and physiological effects in patients with MDD in the real daily clinical practice as an adjunct therapy to the treatment as usual (TAU). Furthermore, we hypothesize that resting heart rate as well as the blood pressure will be lower in the intervention group compared to the control group.

## Methods

### Study design

The present study is a single-centre, open-label and randomized wait-list-controlled trial, which took place at the University Psychiatric Clinics (UPK) Basel (Switzerland) between Mai 2021 and September 2023. For details of the study design see also the study protocol of the trial, published previously [27].

At the baseline, all participants were randomly allocated to two groups / conditions:

1. MBI mobile application “Headspace” + TAU (intervention group)
2. Waitlist + TAU (control group).

**Fig. 1:**
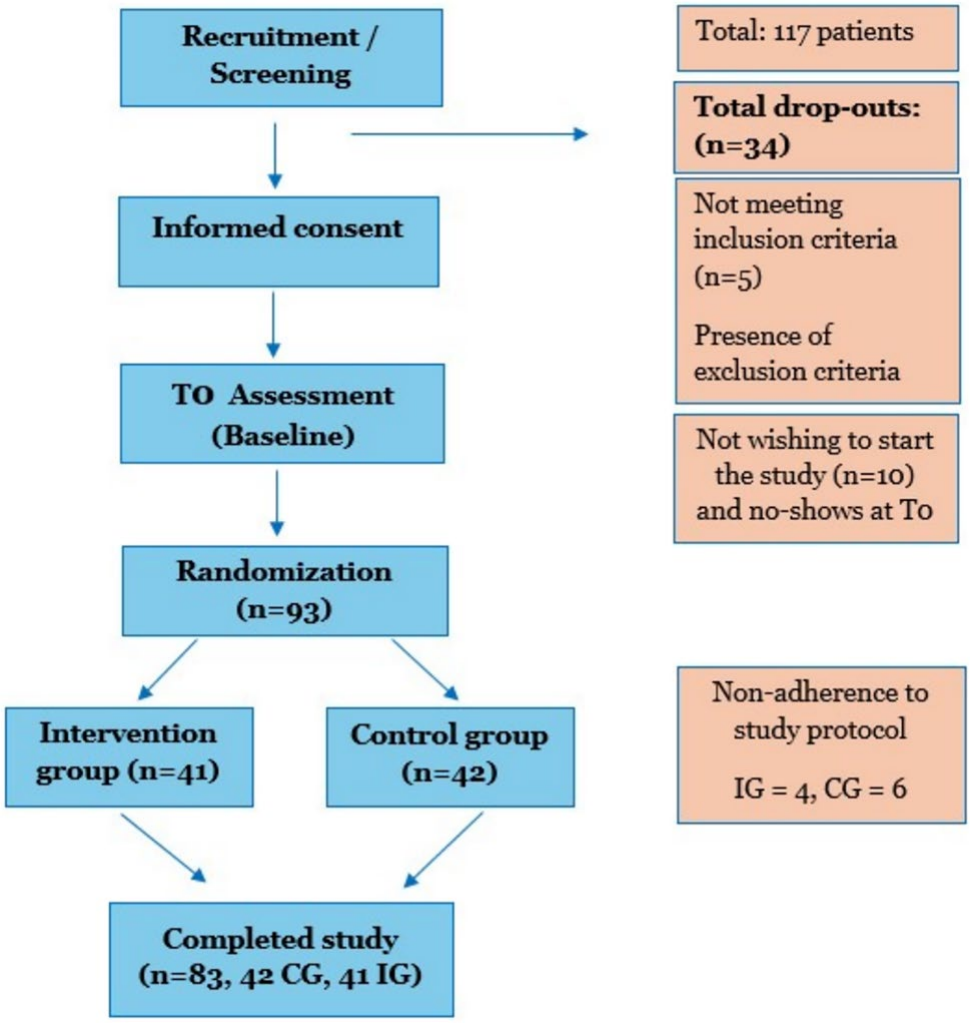
Flowchart study design and drop-outs

In both intervention and control group, two measurements took place: at the baseline (time point T0) and 30 days after baseline (time point T1) (see Fig. 1).

Participants of the control condition had no additional task to their TAU, whereas participants of the intervention group have been introduced to the app “Headspace” and its handling. They were required to use the course “Basics” of the application, which offers 10 guided meditation / mindfulness sessions. The duration of each session can be done in 3, 5 or 10 min. The participants made this course in three series (cycles) of ten days: the first cycle of the course with 3 min per session, the second with 5 min, and the last one with 10 min per session. All participants of the intervention group were asked to do one session a day to a daytime of their choice. To monitor the adherence to the protocol and to keep participants engaged to use the app daily, they received weekly reminder via email.

At T1, the name of the mobile application was communicated also to the participants of the control group so they can use it after the end of the study, if they wished so.

### Primary outcome

#### Symptom severity—objective measurements

To measure the symptom severity, we used the change score of HDRS scores as primary outcome.

The 17-item Hamilton Rating Scale for Depression (HRSD-17) and the Montgomery Asberg Depression Rating Scale (MADRS) are two widely used clinician-rated symptom scales [28]. HDRS, originally published by Max Hamilton in 1960 [29] is since decades an established standard expert-rating scale for depression severity. HDRS exhibited high internal consistency and support for its construct validity was demonstrated by it's patterns of correlations with other measures of depression, anxiety, and depression-relevant cognition and is as a reliable and valid instrument for the assessment of depressive severity [30].

We used the 17-item version of HRDS, which ranges from a minimum of 0 to a maximum of 52 points (including 3-points till 5 points items). HDRS score has been obtained using a standardized interview.

### Secondary outcomes

#### Symptom severity—subjective measurements

The BDI-II is a 21-item self-reporting questionnaire. It evaluates the severity of depression in normal and psychiatric populations [31]. Psychometric analysis conducted by Subica et al. revealed high internal consistency (Cronbach's α = 0.93) and significant (*p* < 0.01) intercorrelations between the BDI-II total scale and Behavior and Symptom Identification Scale-24's Depression/Functioning (r = 0.79) and Overall (r = 0.82) subscales [32].

Authors conclude that BDI-II can be used to measure clinical changes to depressive symptomology over time, however there are limitations of the BDI-II as a diagnostic tool for adult inpatients.

The BDI-II total score can be obtained by adding up the answers of all 21 items, from a minimum of 0 to a maximum of 63 points (total score).

The questionary can be administrated either as a self-rating or interview-based rating scale.

Since the way of administration can lead to different results [33], the self-rating way of administration has been chosen for all participants.

Both BDI-II and HDRS are commonly used psychometric instruments with broad applicability in research and clinical practice [31, 34].

#### Physiological measurements

To assess stress level, resting heart rate, systolic and diastolic blood pressure have been measured by using an automatic blood pressure monitor (with upper arm cuff) provided by the hospital. All measurements were taken in awake subjects in a sitting (slightly inclined) position and in the same room. To reduce the impact of pre-test movements, all subjects were asked to breathe normally and not move for five minutes. The initially aimed assessment of resting respiratory rate has been omitted, since the device to measure respiratory rate (via abdominal and thoracic cuff) has not been delivered on time and counting the taken breath in one minute would not be exact.

#### Consumption of anxiolytics and sedatives, nicotine and alcohol

Furthermore, the self-reported current consumption of alcohol, the consumption of nicotine, in particular number of smoked cigarettes per day as well as the consumption of anxiolytics and sedatives have been assessed on both measurement time points.

Self-reported rehospitalization due to relapse has been assessed at T1 and checked for rehospitalization in the UPK via hospital information system.

### Participants

The previously estimated sample size of over 128 patients, based on a priori power analysis via G*Power [35] for a two-sided *t*-test for independent samples with an expected effect size of Cohen’s d = 0.50 and a power (1 − β) of 80%, could not be reached due to limited personal and financial resources as well as a higher drop-out rate than expected (30% compared to expected 10%).

In total, 117 patients have been recruited. Inclusion criteria were: inpatients or partial inpatients (dayclinic), at least 18 years old, before discharge to ambulatory care, all diagnosed with MDD according to the International Classification of Diseases (ICD-10) with relevant depressive symptoms (BDI > 9 or HDRS > 7 respectively), having their own smartphone or the possibility to use one for the duration of the study. Exclusion criteria were: acute suicidality, dementia, acute substance dependency (two patients excluded), psychotic, schizophrenic or schizoaffective disorders, serious health conditions like unstable cardiovascular, heart, lung, endocrine or neurological disorders, current use of headspace (one patient excluded).

26 patients have been withdrawn from the projects, of which 10 because of non-adherence to the study protocol, 16 did not complete the study (six participants did not show up for the first measure or could not be reached, respectively; and ten at their own request). Thus, the final sample included in the analyses consisted of 83 patients; 41 patients in the intervention condition and 42 patients in the control condition (see Fig. 1). Based on a post hoc power calculation via G*Power [35], the actual power to detect the assumed medium effect of using a one-sided Mann–Whitney U-test for independent samples was 71.3%. As the beta error has no serious consequences, we interpreted a power of 71.3% as acceptable.

**Fig. 2:**
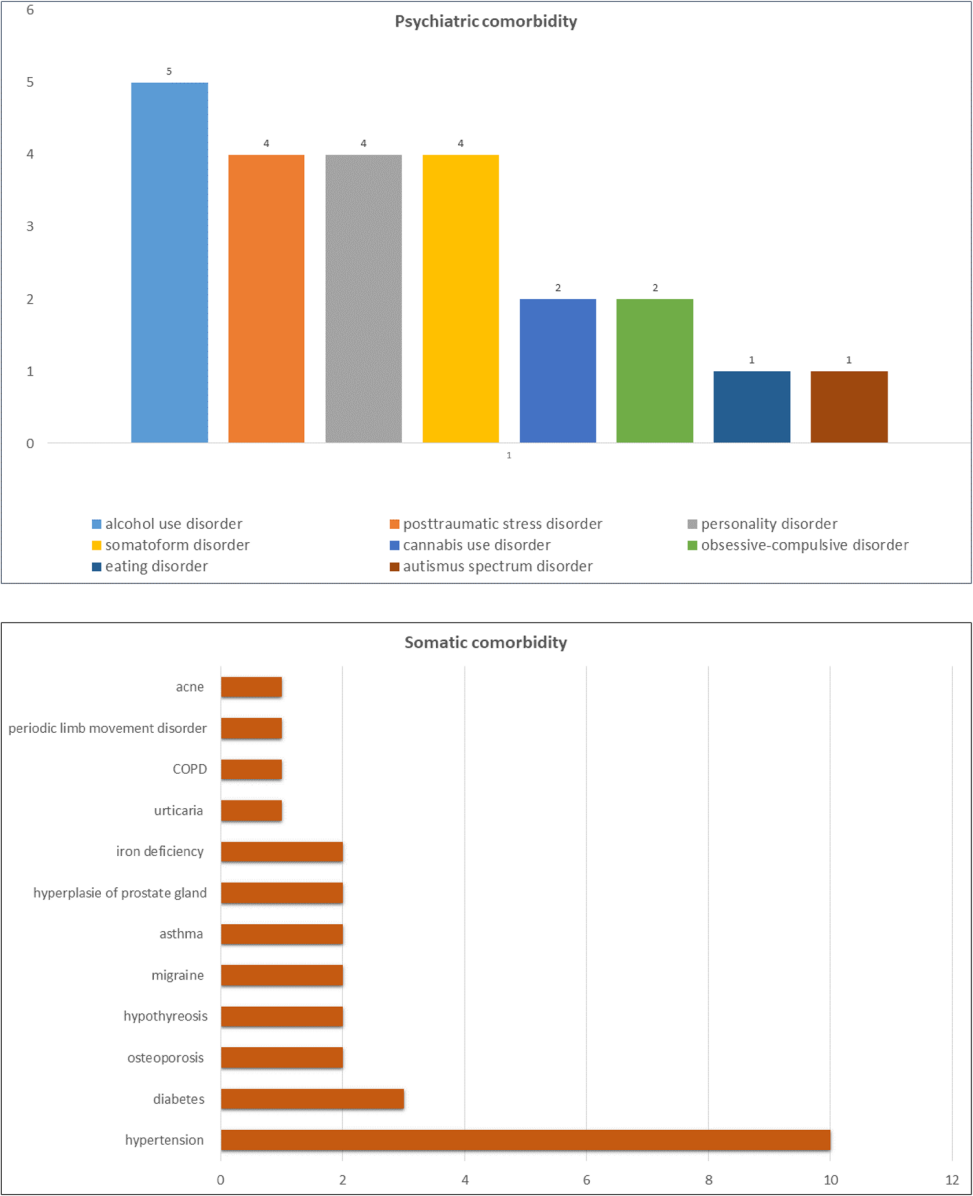
Psychiatric and somatic comorbidities

Of all 83 patients, 47 patients were diagnosed with recurrent depression, 36 patients with a depressive episode as a major diagnose. 18 patients suffered from one (or more) additional psychiatric disorder. For psychiatric and somatic comorbidities see Fig. 2.

#### Medication

**Fig. 3:**
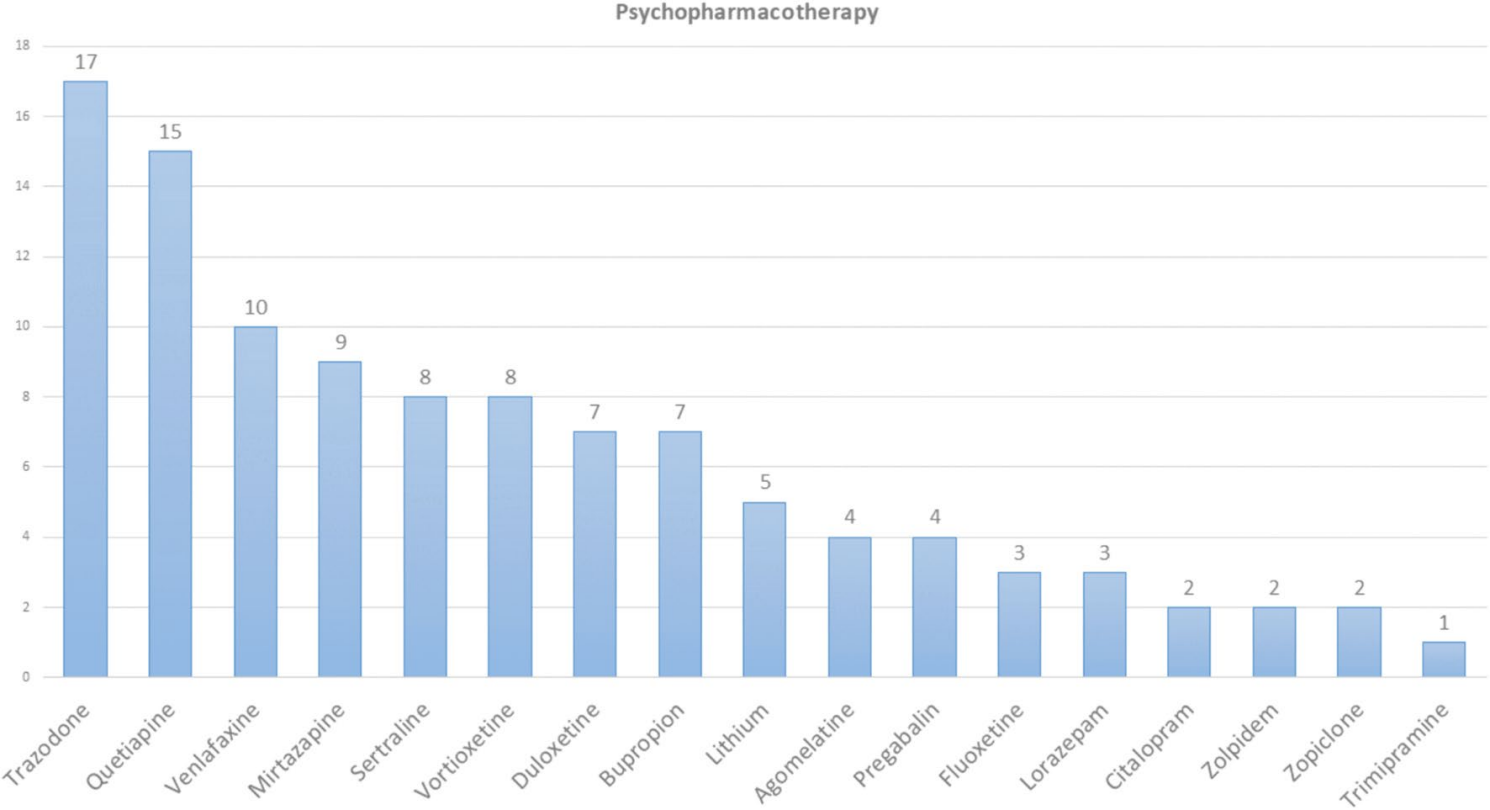
Summary of all psychotropic medication

Eight patients were drug naïve, and 75 were on psychiatric medication. Figure 3 summarizes psychopharmacology taken by all patients.

### Data analysis

Statistical analyses were performed using “R” (version 3.6.3) and following common R-packages: R stats and utils, datasets, formal methods and classes packages [36]. The normality of the distribution was assessed using the Shapiro–Wilk test. To assess differences in change scores between groups, a one-sided independent *t*-test and a Wilcoxon rank-sum test (equivalent to the Mann–Whitney-U-test) for non-normally distributed data were used. Furthermore, the results of the secondary outcomes were Bonferroni corrected to take in account the multiple testing.

## Results

In all measures, at least the change score of one group did not follow the normal distribution. Therefore, we calculated the Mann–Whitney U-test for all our primary and secondary measures.

The mean BDI and HDRS total scores of all patients at the baseline were 15.45 and 11.90 respectively, corresponding with a mild depression. Table 1 summarizes all assessed parameters prior the randomization.

**Table 1:** Basic characteristics of the study population at baseline

Study population: N = 83 Females = 46 (55%) Males = 37 (45%)									
Baseline measurements			All subjects		IG	CG			
	Mean (SD)	Range	Shapiro–Wilk		Mean	Mean	Mann–Whitney-U test		
			W	*p* value			W	*p* value	
Age	41.30 (14.23)	19–73	0.959	0.009	41.93	40.69	878.5	0.866	
Education years	15.13 (3.39)	9.5–25	0.922	< 0.001	15.81	14.78	1039.5	0.099	
BMI	25.23 (4.43)	17–46	0.913	< 0.001	26.05	24.39	673	0.165	
HDRS total score	11.90 (5.46)	5–31	0.091	< 0.001	12.41	11.62	762.5	0.373	
BDI total score	15.45 (9.89)	7–48	0.904	< 0.001	19.43	19.74	851.5	0.938	
Resting heart rate	76.62 (13.18)	52–111	0.958	0.008	77.95	75.33	761	0.366	
Alcohol consumption (units per week)	1.19 (2.98)	0–8	0.042	< 0.001	1.023	1.373	699	0.114	
							*t*-test		
							T	Df	*p* value
Systolic BP	121.4 (13.9)	89–155	0.982	0.257	122.71	120.10	0.856	81	0.395
Diastolic BP	79.3 (9.3)	57–102	0.986	0.539	78.78	79.71	− 0.45	80	0.651

BMI—body mass index; HDRS—Hamilton Depression Rating Scale; BDI—Beck Depression Inventory 2nd version; BP—blood pressure

At baseline, there were no significant differences between control group and intervention group at the baseline in the assessed parameters.

### Group differences

#### Primary outcome: changes of symptom severity after the intervention in HDRS

**Fig. 4:**
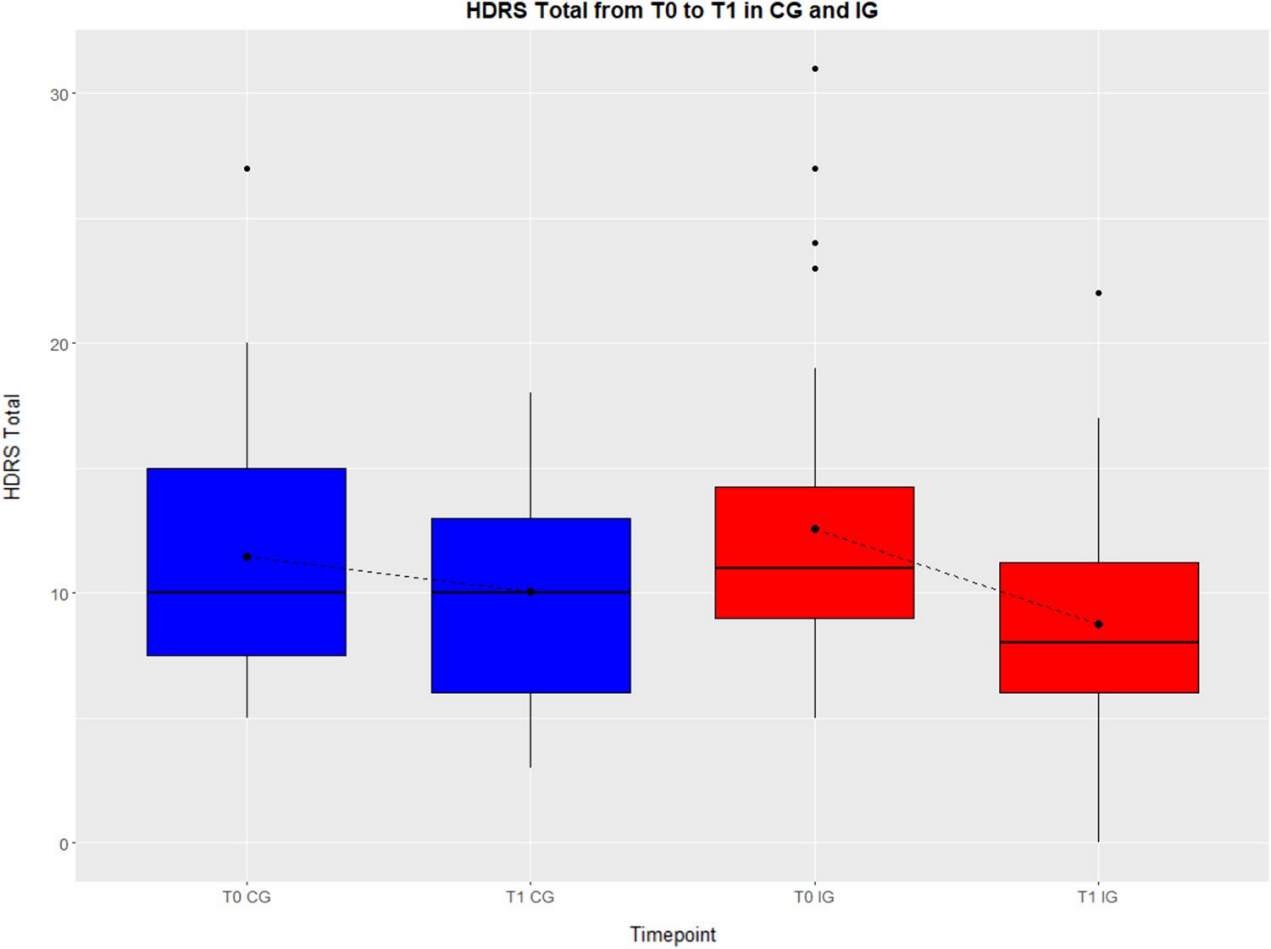
HDRS total score at T0 and T1 in both groups. Bold dot in boxplot = mean

In both groups, HDRS scores were lower at T1 compared to baseline. By applying the Mann–Whitney U test, the change score in HDRS in the intervention group (3.75 points) was significantly higher than the HDRS change score in the control group (1.43 points), *W* = 1095, *p* = 0.033, see also Fig. 4.

In the next step, we conducted an explorative analysis regarding the change scores in every single items of HDRS, to search for a cluster of symptoms that have significantly improved in the intervention group. Items 4 (Early insomnia) and 11 (Physiological concomitants of anxiety) showed significantly higher change score (lower symptom severity at T1) in the intervention group when compared to the control group (Item 4: 0.34 points for IG vs. − 0.05 points for CG, W = 645, *p* = 0.03; Item 11: 0.39 points for IG vs. − 0.05 points for CG, W = 698, *p* = 0.04).

#### Secondary outcomes: changes in subjective symptom severity and physiological measures after the intervention

In accordance to the HDRS change scores, the BDI scores were also lower at T1 than baseline. However, we did not find a significant difference in the change scores between the two groups. Interestingly, in the control group, both BP parameters and the resting heart rate were higher at T1 than at baseline. However the differences between the two groups reached the significance level only for the systolic blood pressure (*p* = 0.0115), see also Table 2.

**Table 2:** Secondary outcomes at baseline (T0) and measurement T1

Secondary outcomes	Baseline (T0)		T1		Change score		Group comparisons	
	IG	CG	IG	CG	IG	CG	Mann–Whitney-U test	
							*W*	*p *value
BDI	19.54	19.73	14.20	16.67	5.34	3.06	684	0.054
Resting heart rate	77.95	75.33	76.34	78.10	1.61	− 2.77	689	0.059
Systolic BP	122.7	122.1	122.3	124.0	0.40	− 1.9	1110	0.0115
Diastolic BP	78.78	79.71	79.17	80.86	− 0.39	− 1.15	854	0.478

IG—intervention group; CG—control group; BDI-II—Beck Depression Inventory II; BP—blood pressure; significance level *p* < 0.0125, Bonferroni corrected

#### Need for hypnotics/tranquilizer

Altogether, seven patients took BZD or hypnotics. One patient from the intervention group reduced her daily BZD dose from 3 to 2 mg lorazepam and one patient from the control group stopped the medication of 0.25 mg lorazepam at T1. Despite a discontinuation of 7.5 mg mirtazapine (one case) as well as discontinuation of 1 g metamizole (one case), the medication remained unchanged.

Tobacco and alcohol consumption: Changes in the nicotine (tobacco) consumption between baseline and T1 could be observed only in six and in alcohol consumption in ten patients, so that the respective analyses have been omitted.

#### Feedback to the application

All patients in the intervention group were asked to provide a feedback about the app use: 33 patients followed this inquiry and commented their experience with the app. 17 patients said they appreciated the app and found it usefull, two patients didn’ t enjoy the app at all. Four patients described difficulties to motivate themselves every day to use the app. Six patients found helpful to use the app always at the same time, with our without reminder. Three patients preferred to use the app at different time during the day. For four patients were the exercises to long (especially the 10 min-cycle), for three patients, on contrary too short. Finally, five patients stated they are motivated to continue with practicing of mindfulness every day, either with help of the app or without.

At T1, the application has been introduced to the control group (waiting list) too. There was no follow-up on the usage of Headspace neither in the control group, nor in the intervention group.

## Discussion

To our knowledge, this is the first randomized controlled study assessing MBI delivered via commercial mobile app on clinical population of patients with MDD. Our results confirmed that MBI delivered via a mobile app can have additional benefits for patients recovering from major depressive episode, compared to treatment as usual.

The intervention group differed from the control group in both self-reported and expert-rating depression severity, however only the difference in the change score in HDRS (primary outcome) was a statistically significant.

Following explanations for the gap between self-reported depression and clinical ratings especially in the recovery process familiar to many clinicians, can be discussed: Comparing the physicians’ and patients’ perspectives regarding the recovery from depressive episode, physicians differ significantly from patients in what they consider important for 'being cured from depression [37]. A meta-analysis conducted by Cuijpers et al. [38] showed significant advantage for ratings done by clinicians when these were directly compared with self-report symptom measures within the same studies. The authors conclude that either self-report measures are more conservative or that clinician-rated improvement is more sensitive to change. It could also be a mixture of both. The fact, that both groups, intervention and control, improved in their depressive symptoms 30 days after baseline, can also be seen in the context of a recovery process lasting weeks or even months after dismission, as often observed by clinicians in patients with partially remission.

Indirect stress level assessment delivered via physiological parameters was an important secondary outcome. Despite the clearly lower resting heart rate and systolic blood pressure in the intervention group, only the difference in the reduction of the systolic BP reached significance.. One possible reason can be the sample size. Furthermore, the effects of mindfulness training on cardiovascular system might be of a different intensity. Although mindfulness can reduce both stress level and depression severity, the way of action in reducing depressive symptoms is complex and not necessarily correlated with the stress reduction [39, 40]. Regarding consumption of alcohol, nicotine and tranquilizer, the conclusions are limited, since only few patients did actually reported changes in consumption of assessed psychotropic substances.

The study has a number of strengths. It is a randomized controlled trial with a sufficient group size. The study population represents a naturalistic patient population dismissed by the psychiatric hospital—MDD patients with comorbidities, mostly on a medication, with some, but not general experience in MBI. The intervention was standardized for each participant in the intervention group.

Following limitations should be mentioned: First, long-term effects are not assessed. Although the intervention of 30 days is within the range of other studies (median = 28 days), we will not be able to measure and report middle- and long-term effects (e.g. 6 months post-intervention). Second, the drop-out rate was relatively high (29%), however comparable to other studies on mindfulness-based interventions in different cohort of patients: (23% [41], 24.5% [8] or 34.5 [42]). Third, the control group was a waiting list only.

## Conclusion

Short-term mindfulness intervention delivered via mobile application seems to be beneficial as adjunctive therapy in patients with partially remitted depressive syndrome.

The adherence can be a considerable limiting factor, especially for middle- or long-term studies as well as for clinical practice.

## Data Availability

Not applicable.

## Abbreviations

BDI-II: Beck depression inventory II
BP: Blood pressure
HDRS: Hamilton depression rating scale
MBI: Mindfulness-based interventions
MBSR: Mindfulness-based stress reduction
MBCT: Mindfulness-based cognitive therapy
MDD: Major depression disorder
TAU: Treatment as usual
UPK: University Psychiatric Clinics
